# When listening to rain sounds boosts arithmetic ability

**DOI:** 10.1371/journal.pone.0192296

**Published:** 2018-02-21

**Authors:** Alice Mado Proverbio, Francesco De Benedetto, Maria Vittoria Ferrari, Giorgia Ferrarini

**Affiliations:** Neuro-Mi Center for Neuroscience, Dept. of Psychology, University of Milano-Bicocca, Milan, Italy; University of Zurich, SWITZERLAND

## Abstract

Studies in the literature have provided conflicting evidence about the effects of background noise or music on concurrent cognitive tasks. Some studies have shown a detrimental effect, while others have shown a beneficial effect of background auditory stimuli. The aim of this study was to investigate the influence of agitating, happy or touching music, as opposed to environmental sounds or silence, on the ability of non-musician subjects to perform arithmetic operations. Fifty university students (25 women and 25 men, 25 introverts and 25 extroverts) volunteered for the study. The participants were administered 180 easy or difficult arithmetic operations (division, multiplication, subtraction and addition) while listening to heavy rain sounds, silence or classical music. Silence was detrimental when participants were faced with difficult arithmetic operations, as it was associated with significantly worse accuracy and slower RTs than music or rain sound conditions. This finding suggests that the benefit of background stimulation was not music-specific but possibly due to an enhanced cerebral alertness level induced by the auditory stimulation. Introverts were always faster than extroverts in solving mathematical problems, except when the latter performed calculations accompanied by the sound of heavy rain, a condition that made them as fast as introverts. While the background auditory stimuli had no effect on the arithmetic ability of either group in the easy condition, it strongly affected extroverts in the difficult condition, with RTs being faster during agitating or joyful music as well as rain sounds, compared to the silent condition. For introverts, agitating music was associated with faster response times than the silent condition. This group difference may be explained on the basis of the notion that introverts have a generally higher arousal level compared to extroverts and would therefore benefit less from the background auditory stimuli.

## Introduction

The aim of this study was to investigate the influence of background music on the ability of non-musician subjects to perform arithmetic operations. For this purpose, the effect of different types of music, environmental noise and silence on the performance of each subject was compared.

Studies that have focused on this matter have taken into account numerous cognitive processes to the extent that the available literature in this area is very heterogeneous and, therefore, difficult to compare. Additionally, the study of this matter has produced contradictory results (see [1] for a review). A meta-analysis performed by Kämpfe and colleagues [2] showed that background music does not have a uniform effect on the performance of tasks. It seems that listening to music has a positive effect on motivational processes and emotional reactions but has a negative effect on cognitive processing (especially memory and language).

For example, listening to music while driving has been shown to improve one’s mood, but it is not clear how it affects performance [3] (van der Zwaag et al., 2012). Beh and Hirst [4] showed that high intensity music decreased performance during high-demand conditions in driving tasks. The positive effect of listening to music on mood has precise neurobiological bases. For example, the fMRI study by Menon and Levitin [5] showed that listening to music modulates the activity of structures involved in the dopaminergic reward system, such as the nucleus accumbens (NAc) and the ventral tegmental area (VTA), which also belong to the circuits of pleasure (see also [6]). Indeed, the so-called *arousal-mood* hypothesis posits that listening to music positively affects task performance by influencing arousal (alertness state) and mood [7].

At this regards an interesting phenomenon has been described, known as the “Mozart effect” [8], for which listening to Mozart’s K448 symphony would enhance spatial reasoning and memory [9], in tasks performed later. Independent meta-analyses and multiple studies found some evidence of the “Mozart effect” [10,11,12], for which visuospatial abilities are improved after 10 minutes of listening to Mozart (or other classical musical pieces). However, it can be speculated that this phenomenon refers to a more general boosting property of music, which has been supported by empirical investigations on the effect of listening to background music on performance during various cognitive tasks. For example, evidence of improvements in verbal memory encoding [13], autobiographical memory in Alzheimer patients [14], declarative memory [15], verbal and visual processing speed [16], arithmetic skills [17], reading [18], and learning second languages [19] has been accumulated. According to other authors [20], there would be no compelling evidence of a special link between listening to Mozart (or to any Classical music) and visuospatial (or spatial-temporal) abilities. Rather, listening to pleasant or preferred music would eventually improve one’s emotional state, thus indirectly influencing cognitive performance.

On the other hand, it is true that background auditory stimuli may negatively interfere with concurrent tasks. According to the *cognitive capacity model*, which was originally advanced by Kahneman’s [21], due to the limited attentional and cognitive resources of our brain when multiple demanding tasks are to be carried out simultaneously (such as listening to music and computing), a general decrease in performance can be observed. For example, Proverbio et al. [22], when observing a group of 54 non-musicians listening to music, nature sounds or silence while studying hundreds of faces, found that background auditory stimuli (rain sounds and joyful music) interfered with memory encoding, which decreased the overall performance. In that study, however, listening to emotionally touching music improved memory for faces and significantly increased heart rate, which was interpreted differently (in the view of a multimodal affective and audiovisual encoding).

Overall, there is solid evidence of a reduction in performance when background music is present [23]. According to Kämpfe et al.’s meta-analysis [2], background music had a small but persistent negative effect on memory performance and related tasks, such as memorizing non-sense syllables or words, especially when music is presented at a loud volume [24,25]; memorizing advertisements (e.g., [26]); and remembering texts read before and after reading (e.g.,[27]). Indeed it has been shown how an intense auditory stimulation might overload perceptual system having distracting effects [28]. Even moderate noise can interfere with cognitive processes and learning [29]. In particular, it has been showed that hippocampal neurons can be damaged by moderate intensity noise exposure, thus resulting in impaired learning and memory abilities [30].

Compared to silence, listening to background music has also been reported to interfere with many other cognitive processes, including arithmetic [31], verbal and numerical tasks [32,33], text reading [34,23], multimedia learning [35], procedural memory [36] and response inhibition in the Stroop task [37]. Considering these findings, it is possible to hypothesize that particularly agitating or complex music might compete for cognitive resources, thus reducing arithmetical performance (as was found in the present study).

Other studies on the effect of music on numerical cognition, however, seem to suggest a boosting effect rather than a detrimental effect. For example, Wolf and Weiner [38] analysed the proportion of correct answers in simple arithmetic problems presented in four different conditions of hearing stimulation: silence, speech, music and industrial noise. The results showed that in the background music condition, the proportion of correct responses was significantly greater than in the condition with industrial noise. Additionally, Hallam and colleagues [39] found that listening to music increased response speed (but not accuracy) during the solving of arithmetic problems that were administered to children aged 10 to 12 years. The authors explained these results using the view of the arousal hypothesis, arguing that music perceived as relaxing had a positive influence on the number of mathematical problems completed and the pro-social behaviour of the children.

In this study, participants were assigned to one of two groups based on their extroversion-introversion traits to test previous hypotheses about inter-individual differences in the responsivity to external noise during attentional focus. Indeed, Eysenck [40], in his personality model, proposed differentiating individuals based on the amount of external stimulation required to reach an optimum level of arousal, linking it to the extraversion personality dimension. According to this theory, the introverted subjects would have an intrinsically high basal arousal level and would thus tend to implement different ways of avoiding stimuli, even those of reduced intensity, to prevent activation from exceeding the perceived optimal level. This model has been supported by electrodermal and electrocortical recording evidence showing that introverts exhibit greater reactivity to sensory stimulation than extraverts (e.g.,[41]). In this regard, Mistry [42] explored the effect of background music amongst extroverts and introverts on test performance via two short comprehension and problem-solving tests and found that extroverts performed significantly better during the completion of the test in the presence of music but performed poorer in silence, while introverts performed better in silent conditions than in the presence of music. The results were discussed in light of Eysenck’s theory [43], according to which extroverts require and prefer highly arousing and stimulating environments to work in to reach their optimum functioning level. Consequently, in Mistry’s [42] study, they extroverts performed better in the presence of music, where their ideal functioning levels were reached. Conversely, Cassidy and MacDonald [8] investigated the effects of music and everyday noise on the performance of introverts and extroverts engaged in the completion of the following five cognitive tasks: immediate recall, free recall, numerical and delayed recall, and the Stroop task. The results showed that performance was lessened across all cognitive tasks in the presence of background sound (music or noise) compared to silence. Performance was modulated by internal arousal, with introverts performing better overall on each task (except the Stroop task) and appearing to be more detrimentally affected by the presence of music and noise.

Here, it was hypothesized that task performance would be modulated by introvert and extrovert tendencies. It was predicted that because introverts’ arousal level is generally higher than that of extroverts, the former would benefit less obviously from an increase in arousal or alertness levels. More generally, we predicted that both groups would be affected by task difficulty and that agitating music was possibly more detrimental than softer music and sounds. Furthermore, it was hypothesized that music boosting effects would be more evident when participants were engaged in solving difficult arithmetical operations.

In this study, only non-musician participants were recruited to avoid the possibility of musicians paying more attention than non-musicians to the musical background and because it has been suggested in the literature that a relationship exists between musicality and numerical ability (e.g., [44,45]).

## Methods

### Participants

Fifty psychology university students (25 women and 25 men), with a mean age of 22.94 yrs. (min = 18, max = 29, SD = 2.49), volunteered for the study. The participants were given academic credits in exchange for their participation. All participants had normal (or corrected-to-normal) vision and normal hearing. All of the participants were right-handed as determined by administration of the Edinburgh laterality questionnaire [46]. Their mean score was 0.72 (scale = -1 left handed, +1 right handed). Fourteen of the participants had a left eye dominance, whereas 26 had a right ocular dominance as determined by administration of 2 practical ocular dominance tests (the *tube* test and the *binocular line alignment* test). The lack of any present or past neurologic or psychic disorder (including epilepsy, acalculia, learning disability disorders, autistic spectrum disorders, and head trauma) was assessed through a self-paced questionnaire. Participants were also administered the Eysenck Extroversion-Introversion scale (*Eysenck Personality Inventory*;[47]) on the basis of which they were divided in two subgroups: those who scored from 1 to 12 were included in the introverts group (25 Ss), while those who scored from 12 to 21 were included in the extroverts group (25 Ss). The psychological profile of the participants was normo-typical, and they only differed in the introversion-extroversion dimension, as can be seen in Table 1.

**Table 1:** Mean scores obtained by the two groups of participants in the three dimensions measured by the *Eysenck Personality Inventory* (Eysenck & Eysenck, 1965): Standard deviation values are reported in italics.

Scale	Max:	Introverts (N = 25)	Extroverts (N = 25)
Extroversion	21	**8** Min = 1; Max = 12 *(3*.*28)*	**16.26** Min = 13; Max = 21 *(2*.*38)*
Psychoticism	25	4.1 Min = 1; Max = 10 *(2*.*4)*	4.03 Min = 1; Max = 10 *(1*.*8)*
Neuroticism	23	11.3 Min = 4; Max = 19 *(3*.*8)*	10.23 Min = 1; Max = 17 *(4*.*3)*

Volunteers were required to refrain from any drug, or heavy alcohol and caffeine consumption within the 24 hours prior to participation. The experiment was conducted with approval from the Ethical Committee of the University of Milano-Bicocca and in compliance with the APA ethical standards for the treatment of human volunteers (1992, American Psychological Association). Informed written consent was obtained from all subjects. All experiments were performed in accordance with the relevant guidelines and regulations.

### Stimuli

Stimuli consisted of a given auditory background (provided through stereo headphones) to be ignored and arithmetical calculations to be solved as fast as possible upon presentation via a PC screen.

#### Auditory stimuli

The stimuli were the same as those used in a previous psychophysiological investigation [22]. Musical pieces were selected according to the procedure described in Proverbio et al. [48]. A group of 20 professional conductors, composers and professors of various Italian conservatories were asked to indicate some representative pieces that best expressed a given emotion. The emotion of a piece was defined as follows: *agitating* if it induced anxiety, distress, fear, agitation, and tension; *happy* if it induced a good mood, wellness, joy, and happiness; and *touching* if it induced pathos, grief, melancholy, pain, sadness, nostalgia, and sympathy. *Tonal* music was defined as any musical production that had a tonal centre around which the melody and harmony were based, including the monodic productions of the Middle Ages. *Atonal* music was defined as any musical production (roughly dated after 1910: from Schönberg onward) that avoided a tonal centre or that used multiple tonal centres simultaneously. A total of 207 suggestions were received: 147 tonal pieces and 60 atonal pieces. The judges were then required to evaluate the corpus on the basis of the 3 emotional categories. The pieces judged as more coherent were then matched for (a) composition and size of the instrumental ensemble, (b) tempo and rhythmic structure, and (c) stylistic distinctiveness (presence of human voices). Pieces that were not comparable along these dimensions were discarded, and in the end, the selected pieces were as follows:

- Donatoni, Franco—Duo pour Bruno (atonal, agitating)
- Bach, Johann Sebastian—St. John Passion BWV 245 (tonal, agitating)
- Part, Arvo—Cantus in memoriam de Benjamin Britten (atonal, touching)
- Bach, Johann Sebastian—II movement from Concerto in D minor for 2 violins (BWV 1043) (tonal, touching)
- Hindemith, Paul—First movement from Kammermusik No. 1 (atonal, joyful)
- Beethoven, Ludwig van—IV Movement of Symphony (tonal, joyful)

Both rain sounds (obtained from an audio file downloaded from the Internet named “75 minutes of thunder and rain—relaxing noise for your ears” https://www.youtube.com/watch?v=WvRv-243Cmk&spfreload=10) and all musical excerpts were cut into 1-minute-long pieces, matched for intensity by means of *MP3Gain* software (89.0 dB), were faded at the end (last second) via *Audacity* software, and were then transformed into MP3 files. The modulation of tonality (used to provide variety) and its possible effect on calculation ability were the factors considered in the present study.

#### Visual stimuli

The visual stimuli consisted of a set of 180 arithmetic operations (division, multiplication, subtraction and addition) that were presented randomly on a PC monitor, and each operation was followed by a hypothetical result, which was correct (right) in half of the cases and was incorrect (wrong) in the other cases. The operations followed these criteria:

- they involved integer numbers from 0 to 2000;
- they could contain up to 8 characters, including the operator;
- they were followed by a result of 0 to 10, which was also an integer number;
- half of them were followed by a correct result, and the other half were followed by an incorrect result; and
- they were all different from each other.

The operations were validated as easy or difficult by 12 graduated judges (5 women and 7 men, aged 26 to 54 years). The judges were asked to evaluate, by means of a Likert 3 point scale, how difficult they found the calculus resolution. The judges were instructed to give a 1 to all arithmetic operations whose result immediately appeared right or wrong, a 2 to all operations for which they had some doubt or uncertainty, and a 3 to all operations that appeared unsolvable. While stimuli rated 3 were eliminated, stimuli rated 1 were considered easy, and stimuli rated 2 were considered difficult. On the basis of this procedure, the level of difficulty was accurately balanced across the correct and incorrect categories. There were 4 categories of stimuli: easy right (N = 45), e.g., [98–98 = 0]; easy wrong (N = 45), e.g., [3x3 = 7]; difficult right (N = 45), e.g., [910:130 = 7]; and difficult wrong (N = 45), e.g., [1862:318 = 9]. Operations were typed in yellow on a bluish grey background and were presented in the centre of the fixation point area. Their maximum eccentricity from the fixation point was 2.5 degrees of the visual angle. The operation result was printed in yellow in a larger font (size = 2 cm) exactly on the fixation point.

### Procedure

The participants comfortably sat in front of a computer screen placed 114 cm from their eyes in an experimental cubicle, which was acoustically and electrically shielded. The subjects were instructed to gaze at the centre of the screen (where a small yellow circle served as a fixation point during the stimulus presentation) and avoid any eye or body movement during the experimental session. The subjects wore a pair of headphones (*Sennheiser* HD 202) for listening to the background auditory stimuli and were instructed to attentively look at the mathematical operation. After the proposed result was flashed, the participants were instructed to press a button with his/her index finger to signal that the response was correct or with his/her middle finger to signal that it was incorrect. The response hand (left or right) was alternated across trials and announced at the beginning of each trial. The sequence order and presentation as well as the response hand order were randomized across subjects. Mathematical operations were presented for 1500 ms and were followed by an inter-stimulus interval (ISI) randomly ranging from 100 to 150 ms. The results of the operations were presented for 1500 ms and were followed by an inter-trial interval (ITI) of 1000 ms. Every experimental sequence was preceded by the presentation of 3 warning signals, “attention”, “set”, and “go”, and each lasted 1 sec with a 1 sec ISI. The experimental session was also preceded by two experimental sequences in which participants practised the response press with both hands and were familiarized with the task procedure. The background auditory stimuli for the training sessions consisted of 2 minutes of nature sounds and Jazz music. Environmental noise consisted of the sound of ocean waves downloaded from *YouTube*, *Google Inc*.: http://bit.ly/1h6jn2D), and Jazz music consisting of an instrumental smooth jazz piece (*Ibiza Piano Bar Music*, *Piano Bar Music Records*, 2013 from *YouTube*, *Google Inc*.: http://bit.ly/1zzafwp).

The experimental sessions consisted of 15-minute runs during which participants had to solve 12 arithmetical operations and immediately decide if the proposed result was right or wrong. Every run included 3 easy correct operations, 3 easy incorrect operations, 3 difficult correct operations and 3 difficult incorrect operations, and the operations were randomly mixed. Of the 15 sequences, 3 were associated with a background of agitating music; 3, with a background of joyful music; 3, with a background of rain sounds; and 3, with silent conditions. Conditions were randomly mixed and mixed across the participants. Half of the subjects listened to music characterized by an atonal style (full of dissonances, dodecaphonic and serial music, such as Boulez’s), while half of them listened to classical tonal music (such as Bach’s).

Experimental sequences were created via the *Eevoke* system (*ASA System*), which controlled stimulus presentation and response recording.

### Data analysis

Response times (RTs) and the percentage of correct responses (hits) were recorded and quantified. RTs that exceeded the mean value ±2 standard deviations were discarded, which resulted in a rejection rate of approximately 5%. Data normality was assessed through the Shapiro-Wilk test (Shapiro-Wilk = 0.97862 for RTs and 0.94816 for hits). Both RTs and accuracy percentages were subjected to separate multifactorial repeated-measures ANOVAs with 2 between-subjects factor and 3 within-subjects factors, whose factors of variability were as follows:

group (extroverts and introverts), tonality condition (tonal or atonal), background (agitating, happy or touching music; rain sounds; or silence), correctness (right or wrong), and difficulty (easy or difficult). Tukey’s post hoc test was used for multiple comparisons among the means. Partial eta squared values were systematically provided to estimate effect sizes. Homoscedasticity was not assumed and p-values were corrected using Greenhouse-Geisser correction.

## Results

### Hits

Overall, the two groups did not differ in their percentage of correct responses (Extroverts: 80.01%, SE = 1.36; Introverts = 80.27%, SE = 1.48), meaning that their mathematical abilities were identical.

**Fig 1:**
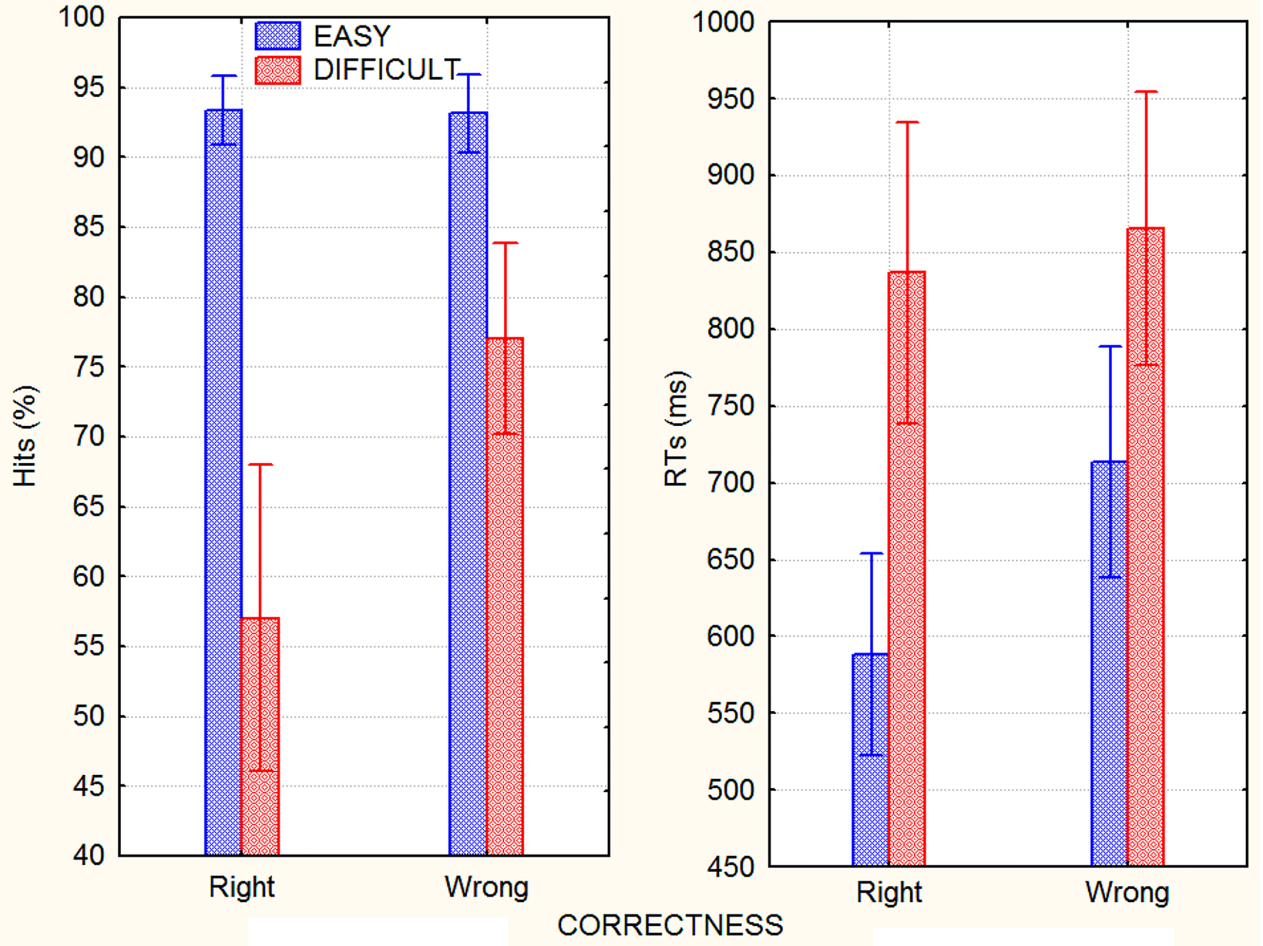
Percentages of correct recognition of right and wrong arithmetic results as a function of operation difficulty (left). Response times relative to the correct recognition of right and wrong arithmetic results as a function of operation difficulty (right).

Musical style (tonal vs. atonal) did not affect performance (p = 0.45). ANOVA of the percentage of correct responses indicated the significance of the background factor [F(4,184) = 16.62; ε = 0.821701; p<0.0001; $\eta_{p}^{2}$ = 0.265]. Relative post hoc comparisons showed that hits were higher for those listening to touching music (84%, SE = 2.082) or rain sounds (82.10%; SE = 2.64) than for those in the silence condition (78.82%, SE = 2.75). The difficulty factor strongly affected performance [F(1,46) = 312.62; ε = 1; p<0.0001; $\eta_{p}^{2}$ = 0.874], with a much larger hit percentage in response to easy (93.3%, SE = 1.6) rather than difficult (67%, SE = 5.35) operations. The interaction of background x difficulty [F(4,184) = 16.12; ε = 0.878456; p<0.0001; $\eta_{p}^{2}$ = 0.26] and relative post hoc comparisons showed that while the background auditory stimuli had no effect on performance during easy calculations, silence (62.2%, SE = 3.26) was associated with a significantly worse performance compared to joyful music (67%, SE = 2.6; p<0.03), touching music (72.16; SE = 2.5; p<0.0001), and rain sounds (70.61%, SE = 2.93; p<0.0001) when dealing with difficult operations. The operations’ correctness strongly affected performance [F(1,46) = 74.14, ε = 1; p<0.0001; $\eta_{p}^{2}$ = 0.63], with higher hits for easy (85%, SE = 2.912) rather than difficult (75,19; SE = 4.3) operations. The interaction of correctness x difficulty [F(1,46) = 77; ε = 1; p<0.0001; $\eta_{p}^{2}$ = 0.63] and relative post hoc comparisons showed that when operations were easy, it was equally difficult to identify correct (93.36%, SE = 1.22) and incorrect results (93.162, SE = 1.39), but when they were difficult, it was easier for participants to recognize incorrect (77.02, SE = 3.384) than correct results (57%, SE = 5.45), as can be seen in Fig 1 (left).

**Fig 2:**
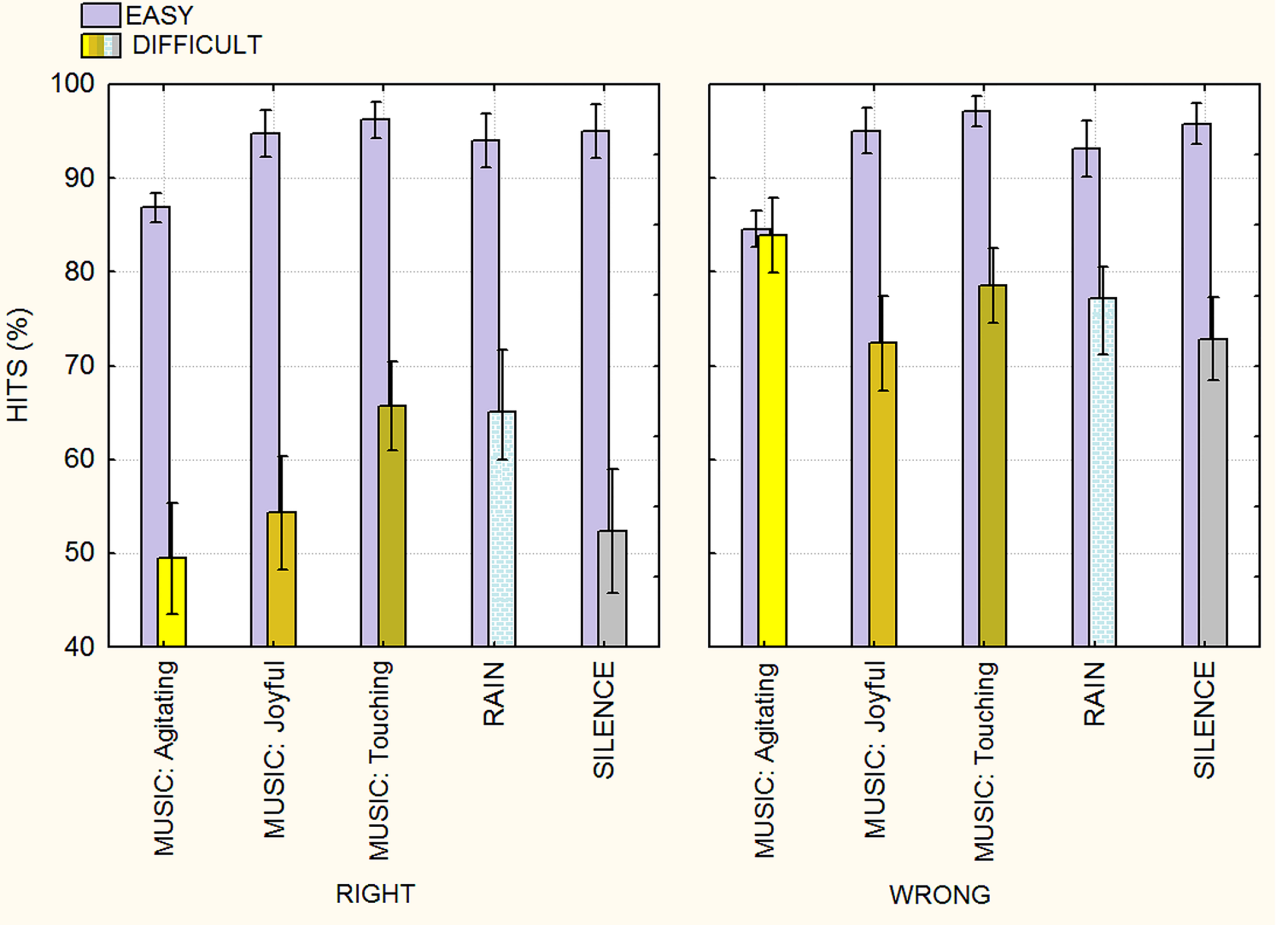
Percentages of correct recognition of right and wrong arithmetic results as a function of operation difficulty and type of background auditory stimuli. There was no effect of the background auditory stimuli on hits during the solving of easy arithmetical operations (the “ceiling effect)”.

The interactions of background x correctness [F(1,184) = 5.78; ε = 0.943212; p<0.0002; $\eta_{p}^{2}$ = 0.12] and relative post-hoc comparisons showed almost a complete lack of background effect during easy calculations. The further interaction of background x correctness x difficulty [F(1,184) = 11.3; ε = 0.928440; p<0.0001; $\eta_{p}^{2}$ = 0.20] was also significant (see Fig 2 for means and standard errors). Post-hoc tests showed a significant increase (p<0.0001) in the percentage of appropriate recognition of difficult right answers during listening to touching music (66%, SE = 2.36) or rain sounds (64%, SE = 3.56) vs. silence (51.6%, SE = 3.4). Only agitating music had the effect of worsening performance during easy calculations, with respect to all background conditions, as shown by post hoc comparisons (p<0.0001). For example, hits were lower when deciding correct results in response to the agitating music (86.86, SE = 0.77) compared to the silent condition (94.99, SE = 1.4) and were lower in the agitating music condition (84.6%, SE = 0.95) than in the silent condition (95.8%, SE = 1.08) when deciding incorrect results (see Fig 2). Atonal happy music was not associated with an improvement in performance when dealing with right answers, as shown by the interaction of background x correctness x style (F(1,184) = 2.68; ε = 0.943212; p<0.04; $\eta_{p}^{2}$ = 0.05).

### Reaction times

The overall response speed ranged from an average of 770 ms (SE = 21.62) for extroverts to an average of 731 ms (SE = 23.49) for introverts. Overall RTs were much faster in response to easy (651 ms, SE = 47.6) than difficult (851 ms, SE = 61.7) operations [F(1, 46) = 2015, ε = 1, p<0.0001; $\eta_{p}^{2}$ = 0.82] and to correct (712.5 ms, SE = 51) than incorrect (790 ms, SE = 54) results [F(1, 46) = 70.454, ε = 1, p = 0.0001; $\eta_{p}^{2}$ = 0.60]. The interaction of style x correctness [F(1, 46) = 9, ε = 1; p<0.005; $\eta_{p}^{2}$ = 0.16] showed slower responses to atonal (818 ms, SE = 77) than tonal incorrect results (761 ms, SE = 76). The interaction between difficulty and correctness [F(1, 46) = 42,385, ε = 1, p = 0.00000; $\eta_{p}^{2}$ = 0.92] and relative post hoc comparisons showed faster responses (p<0.0001) in recognizing right than wrong arithmetical results but in only the easy condition. Means and standard deviations can be seen in Fig 1 (right).

**Fig 3:**
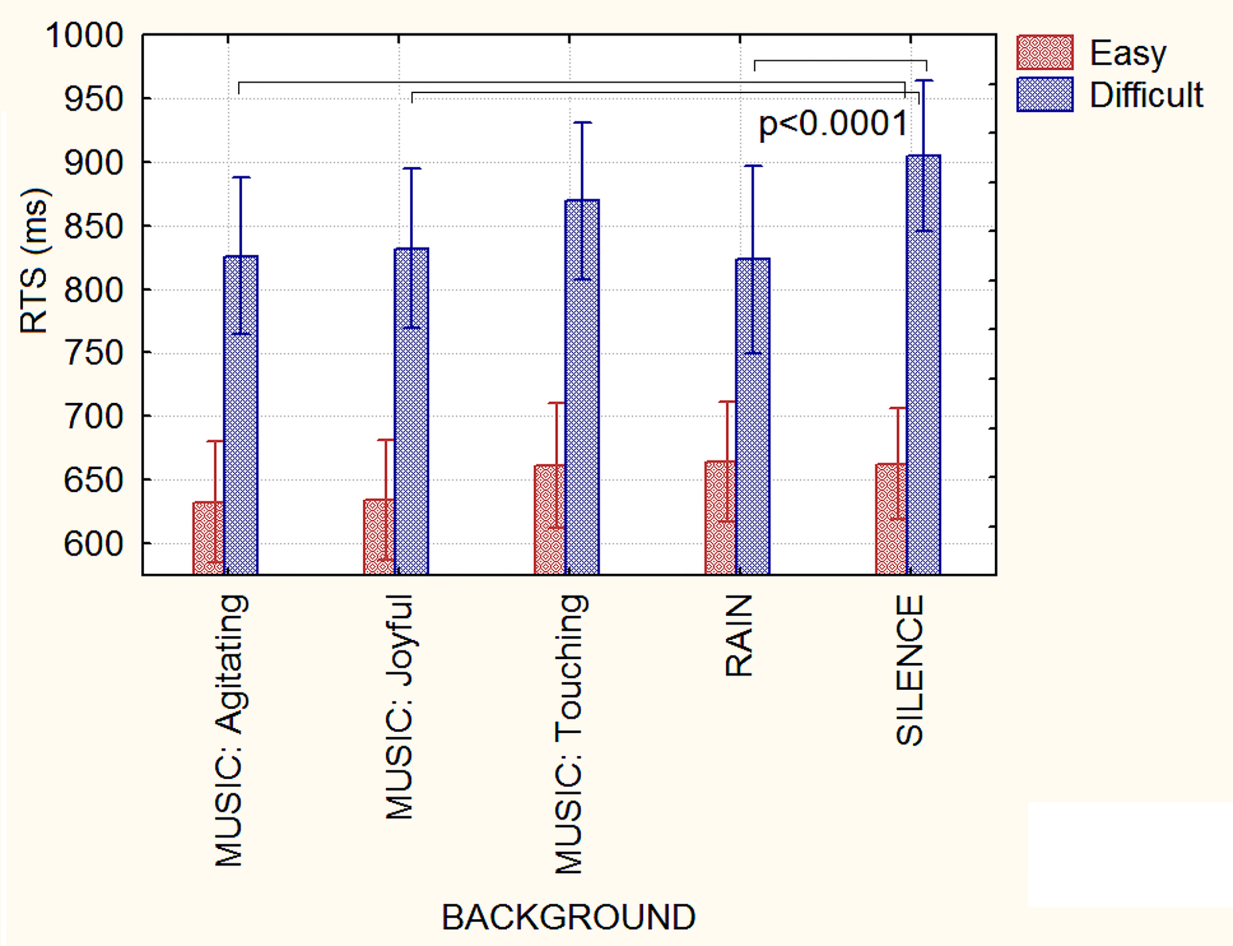
Mean response times (in ms) recorded as a function of the type of background auditory stimuli and operation difficulty. Notably, the background auditory stimuli had no effect on RTs during the solving of easy arithmetical operations (the “floor effect”).

ANOVA yielded the significance of the background factor [F(1,184) = 8.567; ε = 0.850342; p<0.0001; $\eta_{p}^{2}$ = 0.16]. Post hoc comparisons showed slower response times during the silent condition (784 ms, SE = 30.8) than in response to agitating music (p<0.00005, 729 ms, SE = 34) joyful music (p<0.0005, 733 ms, SE = 35) and rain sounds (p<0.001, 744, SE = 39). The interaction of background x difficulty was strongly significant [F(4, 184) = 4.9064, ε = 0.905655; p = 0.001; $\eta_{p}^{2}$ = 0.10]. Post hoc comparisons showed that background auditory stimuli had no effect on RTs in the easy condition. However, RTs were decreased in the difficult conditions in relation to any type of auditory stimulation as opposed to silence (see Fig 3 for means and standard deviations, p<0.0001), except for touching music that tended to be associated with faster RTs (869 ms, SE = 31) than silence (905 ms, SE = 29).

**Fig 4:**
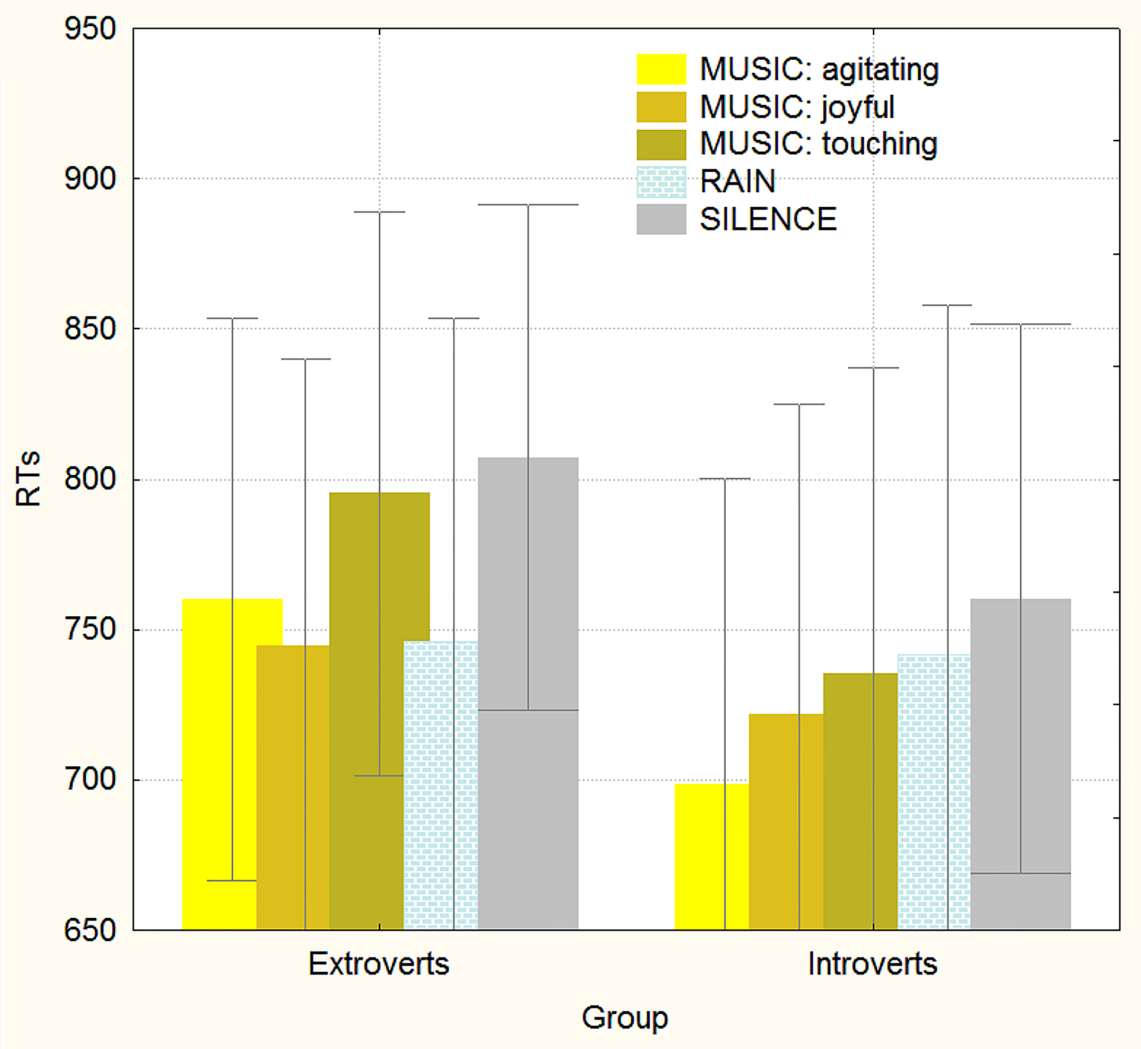
Mean response times (in ms) recorded as a function of group (extroverts vs. introverts) and background auditory stimuli. The auditory stimulation had a stronger “Mozart” (alerting) effect on extroverts.

The significant interaction of group x background [F(4,184) = 2.5; ε = 0.850342; p<0.05; $\eta_{p}^{2}$ = 0.05] and relative post hoc comparisons showed that introverts were always faster than extroverts in solving mathematical problems, except when listening to rain sounds, which made them as fast as introverts (see Fig 4).

**Fig 5:**
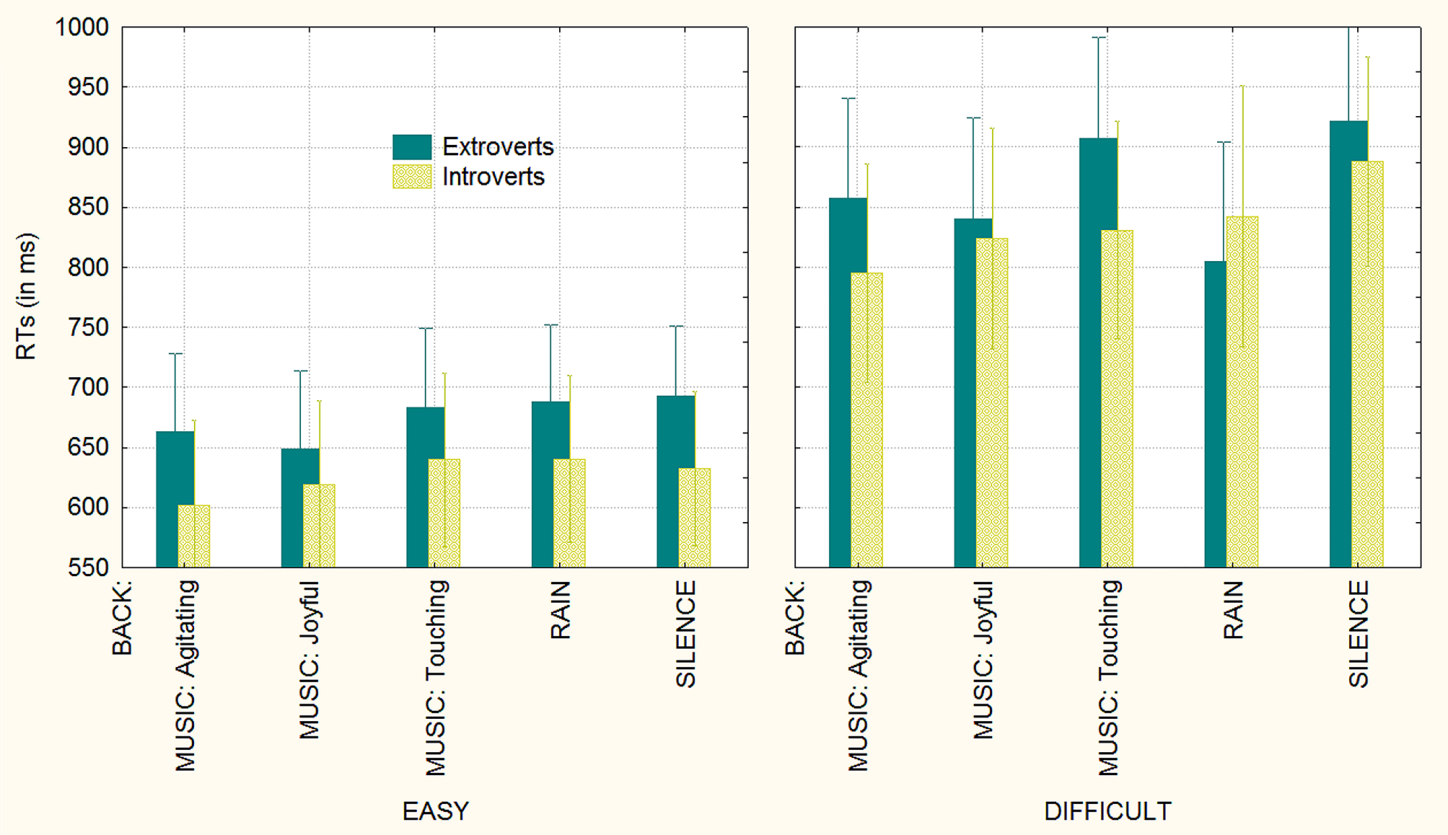
Mean response times (in ms) recorded as a function of group (extroverts vs. introverts), background auditory stimuli and operation difficulty.

The significant triple interaction of group x background x task difficulty [F(4, 184) = 2.6749, ε = 0.905655; p<0.0038; $\eta_{p}^{2}$ = 0.06] and relative post hoc comparisons (see Fig 5 for means and standard deviations) showed that while background auditory stimuli had no effect whatsoever on the arithmetic ability of either group during the easy conditions, it strongly affected extroverts in the difficult condition, with RTs being faster while listening to agitating (857 ms, SE = 42; p<0.027) or joyful music (840 ms, SE = 42; p<0.0006) or to rain sounds (804 ms, SE = 50; p<0.0001), compared to the silent condition (922 ms, SE = 40). For introverts, agitating music was associated with faster (p<0.0009) response times (795 ms, SE = 45) compared to the silent condition (888 ms, SE = 43).

**Fig 6:**
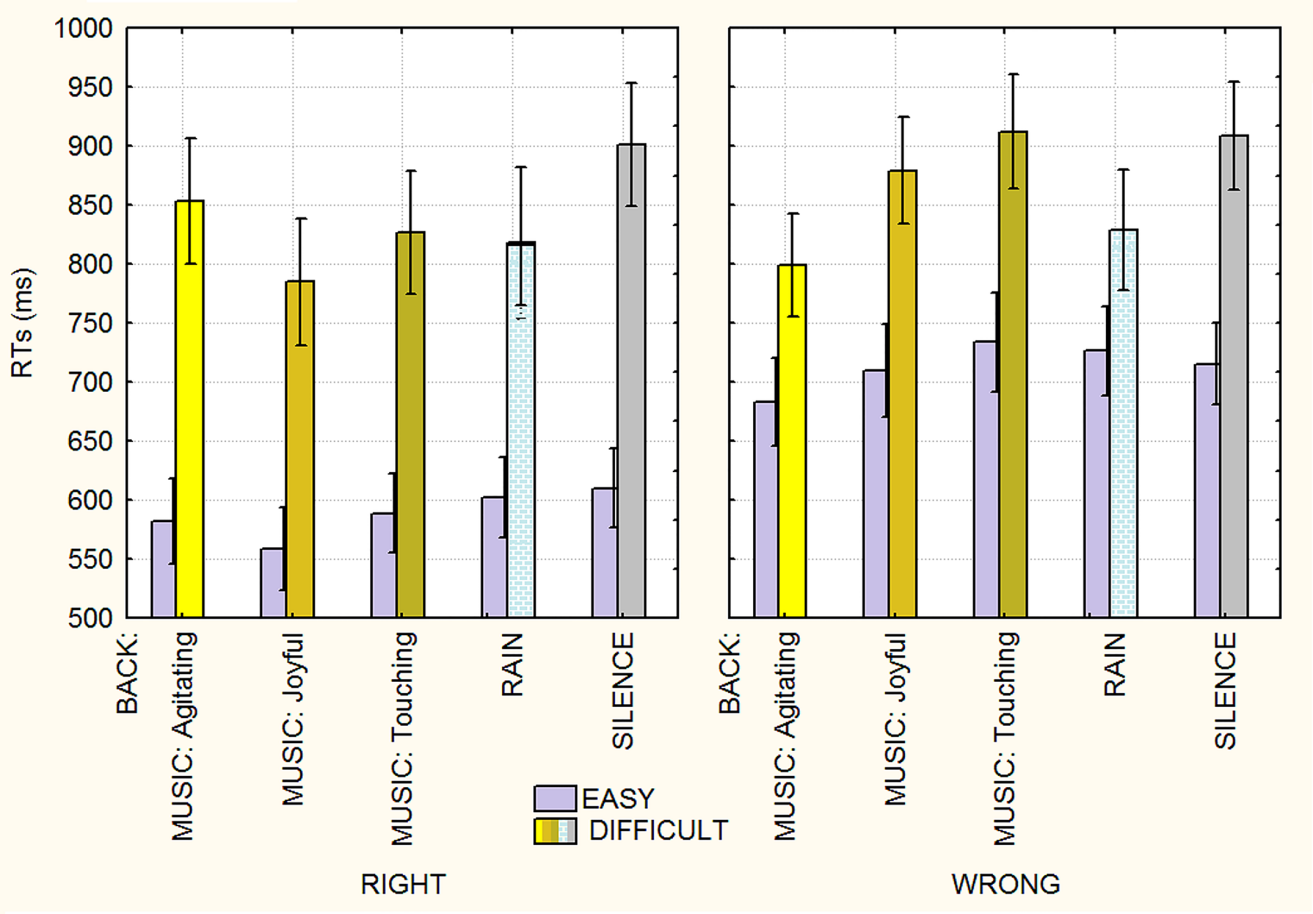
Mean response times (in ms) recorded as a function of background auditory stimuli, result correctness and operation difficulty. Auditory stimulation quickened responses only when the operations were difficult.

Finally, the triple interaction of background x difficulty x correctness [F(4,184) = 2.6; ε = 0.932231; p<0.04; $\eta_{p}^{2}$ = 0.05] showed a significant effect of background auditory stimuli with more difficult conditions. Specifically, post hoc comparisons showed no effect of background auditory stimuli whatsoever on the recognition of correct or incorrect arithmetical results in the easy condition (see Fig 6). For difficult operations, listening to joyful (785 ms, SE = 27, p<0.0001) or touching music (826.5 ms, SE = 26, p<0.008) or to rain sounds (817 ms, SE = 32, p<0.0002) was associated with faster RTs compared to the silent condition (901 ms, SE = 26), with no difference being found between rain or music conditions, when the proposed results were correct. When the proposed results were incorrect, RTs were faster when listening to agitating music (700 ms, SE = 22, p<0.0005) or rain sounds (829 ms, SE = 25, p<0.0006) than in the silent condition (905 ms, SE = 23).

## Discussion

The purpose of this study was to investigate how the presence of musical or environmental background stimuli could influence cognitive processing and, more specifically, arithmetic calculations. In addition, we wished to investigate the possible modulation of this effect due to individual differences in the extroversion/introversion trait.

First, stimulus classification based on the preliminary assessment of the arithmetical stimuli was found to be highly proper: higher accuracy and faster responsiveness were observed for operations classified as easy vs. difficult. Operations also differed on the basis of their correctness but in opposite ways according to the type of variable measured: participants were more accurate in responding to incorrect operations, but they judged the correct results more quickly.

The effect of background on the arithmetical performance was very significant: hits were higher during listening to touching music or rain sounds than during the silent condition. However, the effect was stronger as the task difficulty increased, as predicted by the *cognitive capacity model* [21]. In fact, the beneficial effect of background auditory stimuli was not found when the participants were solving the easy arithmetical operations. Instead, a “ceiling effect” for hits and a “floor effect” for RTs were observed, meaning that the task was too simple to be further facilitated by contextual conditions (such as the background auditory stimuli). Conversely, silence was detrimental when participants were facing difficult arithmetical operations, as it was associated with a significantly worse accuracy than the joyful music, touching music, and rain sound conditions. The interaction with response type (correctness) also indicated a lack of background during listening to touching music or rain in response to easy calculations and a detrimental effect of silence with a greater accuracy in recognizing correct results. This finding can be interpreted in light of the “arousal/mood” hypothesis [8,17], which is not confined to musical material but extended to natural noise (the sound of heavy rain). Indeed, it has been proven that listening to music can boost mood, and white noise seems to have a similar effect on cerebral arousal, as shown by the literature on white noise perception. Ambient, low-level white noise can sound similar to constant rainfall and is a combination of all audible auditory frequencies of equal intensity. In the literature, white noise has been shown to improve the cognitive functioning of children with attention-deficit/hyperactivity disorder [49]. Similarly, a moderate amount of auditory noise has been shown to benefit individuals in hypodopaminergic states [50]. Again, Stansfeld et al. [51] found that road traffic noise can improve performance on episodic memory tasks in children at risk for attentional problems. Therefore, it can be hypothesized that rain noise might increase arousal due to its perceptual similarity to white noise. On the other hand, rain might influence arousal and mood positively due to it being a natural sound. Some authors (e.g.,[52]) have suggested that natural environments have restorative effects following a stressful mental arithmetic task [53] by inducing positive emotional states, but the question needs to be answered via methods such as EEG recordings.

In our study, only agitating music had the effect of worsening performance (with respect to all other conditions) during easy calculations regardless of result type. This finding supports the theory stating that music processing, due to competition with concurrent tasks for access to cognitive resources, would be detrimental to performance. This finding is highly consistent with Kämpfe et al.’s [2] meta-analysis showing how background music has small but persistent negative effects on performance (see also,[24,25]), including arithmetical and numerical tasks [31,32,33].

The data relative to response speed showed faster RTs in response to easy than difficult operations and to correct than incorrect results. The interaction of background x difficulty showed that (similarly to accuracy data) the background had no effect on RTs in the easy condition. Again, quite consistently, RTs were decreased in the difficult conditions by any type of auditory stimulation as opposed to silence, which can be interpreted in light of the arousal hypothesis. Overall, the lack of auditory stimulation during the silent condition was associated with slower RTs than during listening to agitating music, joyful music or rain sounds. This finding suggested that (in our study) the increase in arousal level was not music specific but was due to enhanced cortical excitability induced by the auditory stimulation. In this regard, it has been demonstrated that listening to music may increase EEG frequency and, therefore, cerebral arousal, typically with a modulation of power in the alpha frequency range (e.g.,[54,55,56]).

### Relaxing and agitating music: Neuronal entrainment to beat

It can be hypothesized that the lack of a quickening of RT during listening to touching music, as opposed to other stimulus types, might be due to the slower tempo (e.g., Adagio) that characterized both the tonal and atonal sad pieces with respect to the faster agitating and joyful pieces. While Bach’s melody occurs as some sort of “airy,” relaxed and lyrical dialogue between the two concertante solo violins that alternate and overlap by counterpointing each other (see Proverbio et al. [48] for a complete musicological description of the pieces), in *Cantus in memoriam de Benjamin Britten*, the atmosphere is rarefied and suspended. Conversely, the 1st movement of Kammermusik (by Paul Hindemith, 1922, the opening passage), which was the atonal joyful piece, was characterized by Hindemith himself with the following wording: *Sehrschnell und wild*, translated as "very fast and wild" to refer to the agitated, repetitive and rhythmic nature of the musical writing used. Again, the tonal joyful piece by Beethoven has a fast tempo (Allegro: the last minute of the coda of the 4th movement of Symphony No. 5 in C major, op. 67). The two agitating pieces were “agitating” (distressful, i.e., induced psychological tension and increased anxiety) as described by a group of 20 orchestra directors in Proverbio et al. [22]. Specifically, in Donatoni’s *Duo pour Bruno*, “a state of intense and irrepressible excitement predominates, in which the hinge bar is followed by chord blocks in trill and tremolo by strings, alternated with polyphony of winds. These features can therefore generate a feeling of intense agitation and distress, with furious moments alternated with plaintive states” [48]. The agitating tonal piece, Bach’s St. John Passion in G minor (BWV 245 the opening passage), is also quite dramatic and rhythmic. Conversely, the touching pieces were slower and meditative, characteristics that are relaxing and might reduce the EEG rhythm. In this regard, it is known that while listening to music, the phenomenon of neuronal entrainment to the beat and metre may occur, for which neuronal oscillations in primary sensory cortices may entrain to the attended rhythmic streams [57,58,59,60]. Significant correlations between EEG frequency and the bandpower of the music in the same frequency band over time have been observed [57]. Therefore, a faster auditory beat is associated with a faster EEG frequency, resulting in increased cerebral arousal; this might explain the different pattern of results that were obtained for RTs that were not quickened during listening to touching music (meditative and slow tempo) with respect to the silent condition, as they were with other music types, including heavy rain.

### Introversion-extroversion trait and alertness state

The significant interaction of group per background showed that introverts were always faster than extroverts in solving mathematical problems, except when listening to rain sounds, a condition that made them as fast as introverts. Furthermore, while the background auditory stimuli had no effect whatsoever on the arithmetic ability of either group in the easy condition, it strongly affected extroverts in the difficult condition, with RTs being faster during listening to agitating or joyful music as well as to rain sounds compared to the silent condition. For introverts, agitating music was associated with faster response times than the silent condition. Therefore, overall, the background auditory stimuli increased cerebral arousal more so in extroverts than in introverts. This finding may be explained on the basis of the notion that introverts have a generally higher arousal level compared to extroverts [61,62]; therefore, they would benefit less from the auditory background. For example, this notion has been demonstrated by electrophysiological studies providing evidence that introverts elicit larger sensory (N1)-evoked responses compared to extroverts in response to auditory tones (e.g.,[61, 63, 64, 65, 66]). In our study, introverts were generally faster than extroverts in making calculations. This piece of data may be considered in two ways: either according to the theory that central processes associated with stimulus analysis are faster and, thus, more efficient in introverts than in extraverts, as supported by some authors [67], or to the view that introverts are faster than extroverts in tasks assessing response speed and motor control [68,69] possibly because of a more elaborate analysis of sensory information for introverts. Nevertheless, the literature has shown that introverts are more rapid than extroverts in processing information at the premotor level, which demonstrates that, if necessary, introverts are capable of analysing stimuli more quickly than extroverted individuals (e.g., [67]).

Last, the dissociation between accuracy and response speed (i.e., the lack of a group effect for the former set of data) shares some similarity with what was reported by Hallam and colleagues [39], who found that listening to music increased the response speed (but not accuracy) during the solving of arithmetic problems. Here, we actually did find background effects on accuracy, though less consistent and, more importantly, not articulated as a function of group. This finding suggests that the background auditory stimuli had either the power to increase cerebral arousal, resulting in faster responses, or to improve mental concentration, resulting in greater accuracy, and not affecting mathematical ability per se. In an interesting pioneer study, it was shown that background music increased the percentage of target detection in a visual vigilance task, when conditions were more difficult [70], which suggested aspecific modulation of the alertness state caused by listening to music.

Overall, the effects of background music on cognition depend on different variables: it can facilitate, impair, or have no effect on performance. Variables that seem to play a role range from individual differences (e.g., Introversion-Extroversion trait, musical expertise or music preferences) the type of concurrent task performed, and the type of background music used in the study, in interaction with the listener preferences; e.g. pop vs. Classical music [71, 20].

## Conclusions

In summary, we believe that it may be concluded that apart from specifications—(e.g., extroverts benefiting more because of their lower basic arousal level [62], touching music failing to boost performance and RTs due to its relaxing properties and slow tempo [72], agitating music being detrimental to accuracy during easy calculations because it interfered and overloaded attentional capacities [2], etc.)—a background auditory stimulus consisting of high tempo, unfamiliar classical music pieces, as well as rain sounds (a storm with intense rain and occasional thunder), improved performance and quickened RTs during difficult arithmetic calculations. It can be speculated that the auditory background increased cerebral arousal and alertness levels [72]. It should be noted that this conclusion strictly refers to a non-musician population not particularly drawn to or distracted by musical material.

One study limit is the lack of EEG measures associated with the behavioural performance, which might have provided direct data on the brain arousal level, but a current study in our lab is addressing this specific issue. The other possible limit is that we did not systematically compare and modulate the music or rain tempo to reduce/increase cerebral arousal because music pieces were selected on the basis of a previous psychophysiological study aimed at investigating the effect of the emotional content of the pieces on SNA response (not its metric structure).
